# Miniaturized Lens Antenna with Enhanced Gain and Dual-Focusing for Millimeter-Wave Radar System

**DOI:** 10.3390/mi15030335

**Published:** 2024-02-28

**Authors:** Jian Wang, Junping Duan, Xinxin Shen, Yongsheng Wang, Binzhen Zhang

**Affiliations:** State Key Laboratory of Dynamic Measurement Technology, North University of China, Taiyuan 030051, China; s202106004@st.nuc.edu.cn (J.W.); b20230603@st.nuc.edu.cn (X.S.); b20230612@st.nuc.edu.cn (Y.W.); zhangbinzhen@nuc.edu.cn (B.Z.)

**Keywords:** waveguide antenna, miniaturization, dual-focusing, radome, gain enhancement, UV-LIGA technology

## Abstract

This paper presents a waveguide Lens antenna at the W-band adopting dual-focusing Lens to improve the performance. The Lens antenna consisted of a waveguide slotted structure and lenses processed using NOA73 meet the demands of miniaturization for current communication systems. The antenna radome fabricated using NOA73 not only protects the antenna structure but also improves the gain of the antenna by about 9.5 dBi via electromagnetic wave dual-focusing. A prototype is fabricated using novel UV-LIGA technology. Measured results are compared with simulated values. Measured results confirmed the fabricated antenna operated in the W-band with a 10 dB fractional bandwidth (FBW) of 6.5% from 97.5 to 104 GHz and a peak gain of 22 dBi at 100 GHz in the direction perpendicular to the plane of the feed waveguide. A good agreement between simulation and measurement is obtained, demonstrating efficient radiations in the operating band.

## 1. Introduction

The antenna presented excellent communication ability when working at the millimeter wave (MMW) frequency band. It involves wide-ranging advantages including strong anti-interference ability [1,2], small structure size, high precision and high target-recognition resolution [3], good communication security at night and radiation efficiency with a more stable gain within the wide bandwidth [4]. The antenna in the W-band may be used in airborne, spaceborne, or aircraft collision-avoidance radars [5]; civil avoidance security; passive millimeter wave imaging; and radar sensors [6,7]. Some kinds of antennas have been researched for MMW wireless communication, such as slotted waveguide antennas [8], coplanar waveguide antennas, horn antennas [9], flared SIW antennas, and Lens antennas [10]. The Lens waveguide has been researched for its applications in microwave antennas for many years [11,12,13]. However, few researched antennas can reach a high radiation gain of 22 dBi at the W-band without using antenna array. The Lens antenna designed by F. Nsengiyumva et al. uses MSLA 3D printing technology, with a gain of only 13.74–18.8. The Luneburg Lens antenna designed by Z. Larimore et al. achieved a gain of 24 dBi, but it sacrificed miniaturization, and the array design increased the complexity of the antenna and increased production costs. The selection of Lens structure and materials has a significant impact on the overall performance of antennas [14,15].The Lens antenna will follow the convergent and divergent characteristic for higher frequency applications.

The most critical parts of a radar system are low-cost and lightweight antenna components. Several types of preparation methods have been studied for MMW antennas, such as 3D-printed plastic, LTCC [16], and BiCMOS Operating [17] precision machining [18]. LTCC and BiCMOS technology are complex and expensive processes, 3D-printed technology is limited by materials. In addition, the metal devices fabricated using precision machining technologies were too heavy to meet the light-weight demand in modern wireless systems. The MEMS process has received extensive attention for its micron-level accuracy to meet the requirements of THz devices. Considering the demand of light-weight devices, miniaturization devices, and high-precision, high-frequency devices, this design is based on UV lithography (UV-LIGA) technology.

In this paper, a novel Lens antenna with a high efficiency is proposed. The different center frequency is realized by varying the radius of Lens and the Lens antenna with a radius of 503 µm has been fabricated to validate the effectiveness of the antenna. One of the most striking features of this structure is the use of a new low dielectric constant material NOA73 to make lenses, which has increased the gain of antennas from 12.5 dBi to 22 dBi. It is fabricated using the UV-LIGA technology manufacturing process, which has solved the problem of high weight and high cost. The major structure is implemented using SU-8 photoresist. The fabrication process is easier and the cost of the polymer antenna is greatly reduced. The measured maximum gain of the Lens antenna is 22 dBi with a center frequency of 100 GHz. Compared with the regular SIW slot and microstrip array antennas, the proposed antenna has high gain and small size. The proposed antenna provides a new method idea for millimeter wave radar systems.

## 2. Materials and Methods

**Figure 1:**
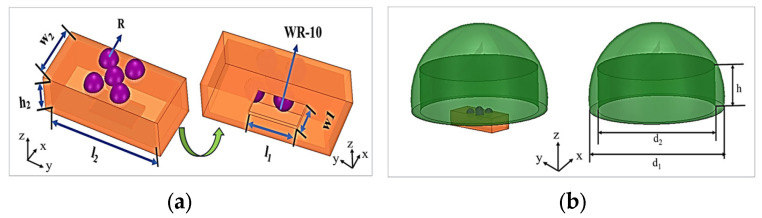
The structure of the Lens antenna: (**a**) Lens antenna; (**b**) Lens antenna with radome.

The antenna structure proposed in this paper and its specific sizes are shown in Figure 1. The antenna structure is composed of a large rectangular cavity. Five circular slot structures are embedded on the upper surface of the rectangular cavity for electromagnetic wave radiation. Five NOA73 spherical lenses (SUNNY OPTICAL TECHNOLOGY (GROUP) Co., Ltd., Yuyao, China) are embedded in the circular holes of the rectangular cavity for preliminary focusing of electromagnetic waves. The corresponding width and height of the waveguide feeding ports are designed to be l_1_ = 2.54 mm and w_1_ = 1.27 mm, and the sizes of the radiation waveguide are l_2_ = 6 mm, w_2_ = 2.54 mm, and h_2_ = 1.27 mm, respectively. The circular radiating port radius is set to R = 0.503 mm. A radome is placed above the waveguide Lens antenna to improve the gain and directivity of the antenna via electromagnetic wave dual-focusing.

### 2.1. Design of Radiation Waveguide

**Figure 2:**
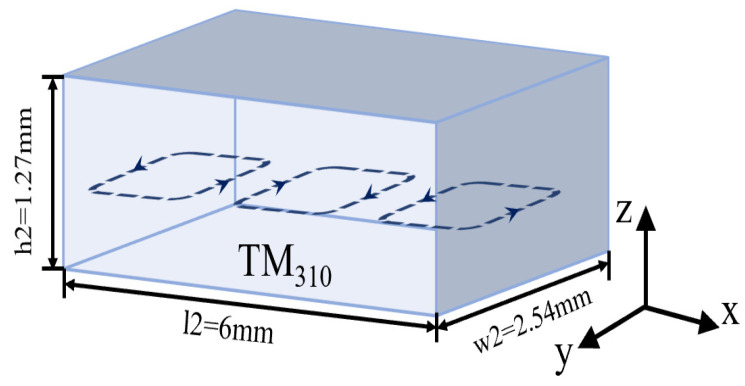
Magnetic field patterns of the resonant modes inside the oversized cavity.

The waveguide radiation structure is composed of an oversized rectangular cavity, which consists of a main TM310 mode cavity and five circular radiation apertures at the top of the waveguide. It has a relatively larger cavity that can easily reduce the implementation sensitivity for the W-band. The WR-10 feeding port is designed to be located at the bottom of the waveguide. The antenna can provide over double the radiation slots needed to achieve a higher element gain using a simpler structure. The magnetic field of the mode is depicted in Figure 2.

For a wave to propagate through a waveguide it is required to be above the cut-off frequency and can be carried out by using the well-known equation

(1) $$f_{c}=\frac{c}{2}\sqrt{{\left(\frac{m}{a}\right)}^{2}+{\left(\frac{n}{b}\right)}^{2}}$$

where *a* and *b* are dimensions along *x* and *y* axes, respectively, *c* is the speed of light in a vacuum, and the integers *m*, *n* > 0 are mode numbers.

**Figure 3:**
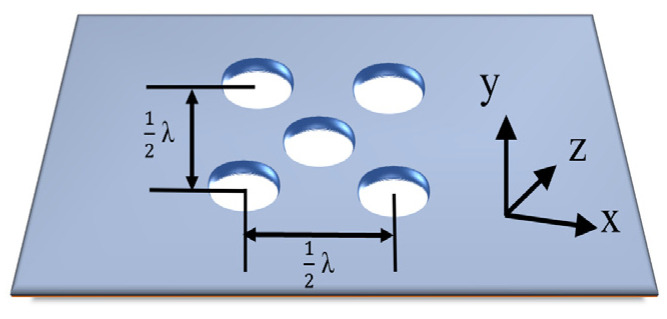
Configuration of the slot array.

To enable the waveguide to achieve radiation, the slots are added to the top surface of the waveguide. The locations of the five radiation slots are shown in Figure 3. One of the slots is in the center of the radiation surface and others are divided into two groups along the *x*-axis and *y*-axis with a space of half a wavelength and they are placed a quarter of a wavelength far from the middle slot to achieve an in-phase distribution. To support a uniform distribution, all radiation slots have the same dimension. Due to the effect of the feeding structure, the electromagnetic field distribution is not as exactly uniform as expected. And, the optimum size of the antenna is decided using ANSYS Electronics Desktop 2017 software, version 23.2.

**Figure 4:**
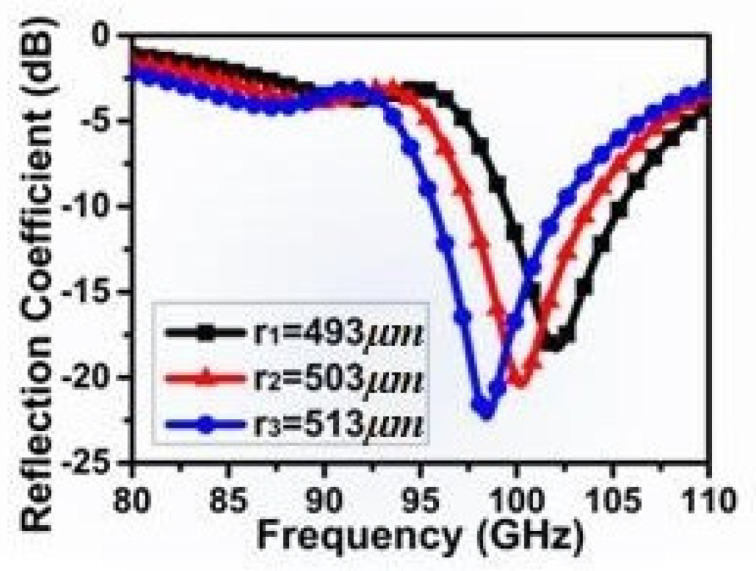
The simulated reflection coefficient of the antenna.

Simulation results of the reflection coefficient of the aperture antenna with different radiuses has shown that the center frequency decreases with the increasing diameter of circular aperture in Figure 4. This parameter can be used to tune the resonant frequency of the antenna and recognize the size of the circular aperture. Therefore, this provides a new method to detect molecules adopting antennas of higher frequency. When adapting the diameter of 503 µm and the center frequency of 100 GHz for research, the bandwidth can reach 8 GHz.

### 2.2. Design of Dual-Focusing LENSES

**Figure 5:**
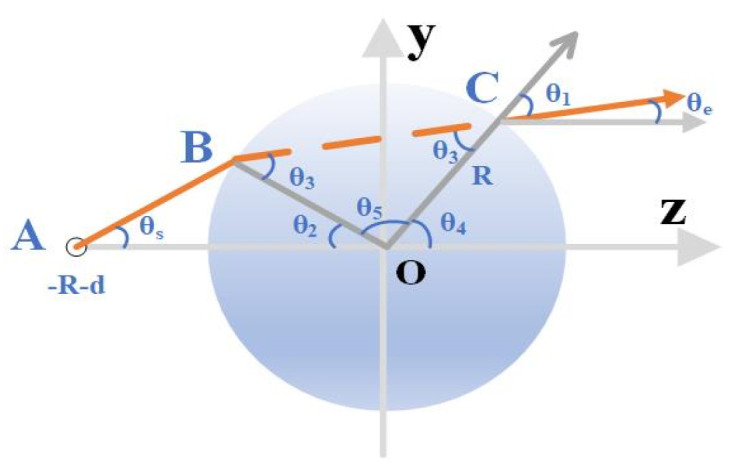
Electromagnetic wave propagation path diagram.

To improve the directivity of the antenna, a dielectric Lens is loaded at the circular aperture to focus the electromagnetic wave initially. For the MM-Wave, it can refract electromagnetic waves from the feed port into parallel waves with equal phase for an ideal Lens, as shown in Figure 5. Conversely, electromagnetic waves can also pass through the other side of the Lens and focus on a single point. The electromagnetic waves passing through the Lens antenna are refracted twice, so the spherical wavefront generated by the primary feeding antenna is converted to an approximate plane wavefront.

The geometry of the electromagnetic wave transmission in the Lens has been shown in Figure 5 and the combined ray-optics method has been used as follows. In this case, before escaping out of the spherical dielectric Lens, each ray will go through double refraction and be divided into three parts. Two refractions occur when rays enter and exit the Lens, respectively, at the points of B and C in Figure 3. Then, we start to track the rays. Firstly, the angle of incidence of the ray *θ*_s_ can be defined using Formula (2) [19]:

(2) $$\theta_{s}\leq \theta_{m}=arc\sin\frac{R}{d+R}$$

where $\theta_{m}$ represents the maximum value of the angle of incidence and *d* is the minimum value of the distance from the feeding port to the surface of the Lens. The other angles in Figure 5 can be calculated as follows:

(3) $$\theta_{1}={\sin(\frac{\sin\theta_{s}}{\sin\theta_{m}})}^{-1},$$

(4) $$\theta_{2}=\theta_{1}-\theta_{s}.$$

According to the Snell’s law, the refractive index in a vacuum is 1 and let the refractive index in the Lens be *n*. Then, the refractive index in the Lens can be expressed as [20]

(5) $$n=\frac{\sin\theta_{1}}{\sin\theta_{3}}.$$

Then, $\theta_{3}$ and $\theta_{4}$ can be obtained as follows:

(6) $$\theta_{3}=arc\sin\frac{\sin\theta_{1}}{n},$$

(7) $$\theta_{4}=2\theta_{3}-\theta_{2},$$

(8) $$\theta_{5}=\pi -2\theta_{3},$$

(9) $$\theta_{e}=\theta_{4}-\theta_{1}.$$

**Figure 6:**
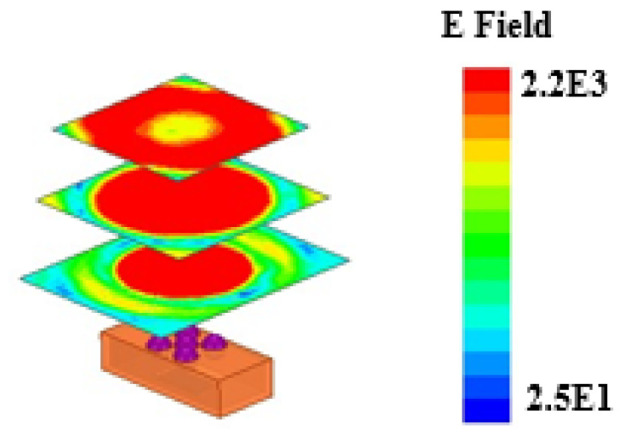
Focusing properties shown with an incoming plane wave of a Lens seen from electric field computation.

The designed waveguide Lens structure has a *d*/*R* of about 1.67. The feed port of the Lens antenna is not at the focus of the Lens, according to the ray diffraction method. As a result, the loaded Lens can only focus the electromagnetic wave to a certain extent while making it completely collimated. Electromagnetic simulation software based on the finite element method is adopted in the simulation procedure. This method can be used to solve cavity problems and antenna coupling precisely. The electric field distribution calculated using the electromagnetic simulation software is shown in Figure 6.

In order to focus the electromagnetic waves and improve the performance of the whole antenna, we adopt the dual-focused methods to greatly enhance the gain of the Lens antenna and realize beam scanning. The radome is designed as a semispherical structure with a cylinder dug out of the inside in Figure 1. The size of the radome is d_1_ = 16 mm, d_2_ = 13 mm, and h = 4.5 mm. The radome will become a new method to improve the performance of the antenna, protect the antenna, and prolong the antenna’s life.

**Figure 7:**
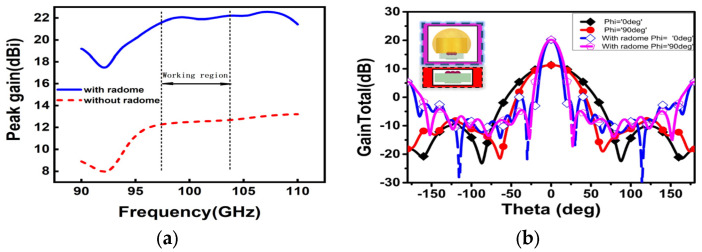
Simulated results: (**a**) Peak gain; (**b**) Simulated radiation pattern in 100 GHz.

**Figure 8:**
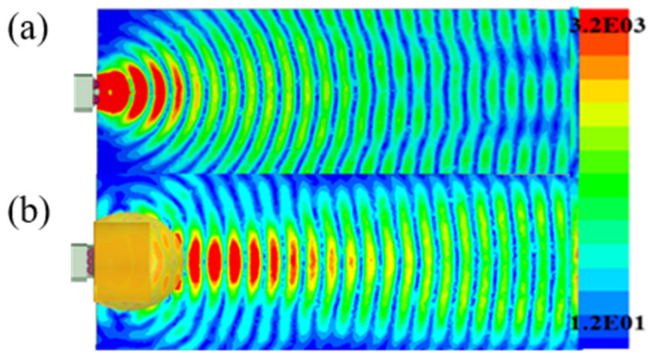
The near-field amplitude distribution in the E plane: (**a**) The near-field amplitude distribution without the radome; (**b**) The near-field amplitude distribution with the radome.

The antenna radome can refocus the electromagnetic wave to realize the enhancement of gain again. According to the finite element method, the performance of the dielectric Lens antenna has improved through the reasonable design and the simulation of the antenna radome. The whole structure is simulated and optimized via electromagnetic simulation software. The simulated result in Figure 7a shows the comparation of peak gain in the working region without the radome and with the radome. The result has shown that the peak gain has great improvement around 10 ± 0.5 dBi in the working region. In addition, Figure 7b has shown that without the radome the half power beamwidth is 46°, while adding the Lens radome over the antenna reduces the half power beamwidth to 17°. Without the radome, the gain in the Lens antenna is close to 12.5 dBi at 100 GHz. The simulation’s gain increases to around 22 dBi after the radome is added. The main lobe width is reduced and the antenna radiation is concentrated, thereby improving directivity, as shown in Figure 8. Furthermore, this Lens antenna has a lower first sidelobe of −5 dBi in the E plane and 0 dBi in the H plane. As a result, the Lens radome reduces the impact of the outdoor environment on its use.

We know that using the radome has increased the use time of antennas, but the use of the radome will reduce the antenna’s performance [21]. Using this method, we can not only protect the structure of the antenna but also improve the performance of the antenna.

### 2.3. MEMS Processing Method and Preparation Process

The dimensions of the high frequency device become smaller with the increasing frequency. Although many current technologies such as 3D printing and computer numerical control machining (CNC) can reach micro-level accuracy, they still have the disadvantages of high processing costs, heavy processing devices, etc. The W-band waveguide antenna is micron-sized; the required fabrication accuracy cannot be met using conventional mechanical processing technology [22,23]. UV-LIGA technology not only has the advantage of high precision but also has the advantage of light weight, etc., which overcomes the problems existing in other processes. Therefore, it was used to fabricate the waveguide antenna.

**Figure 9:**
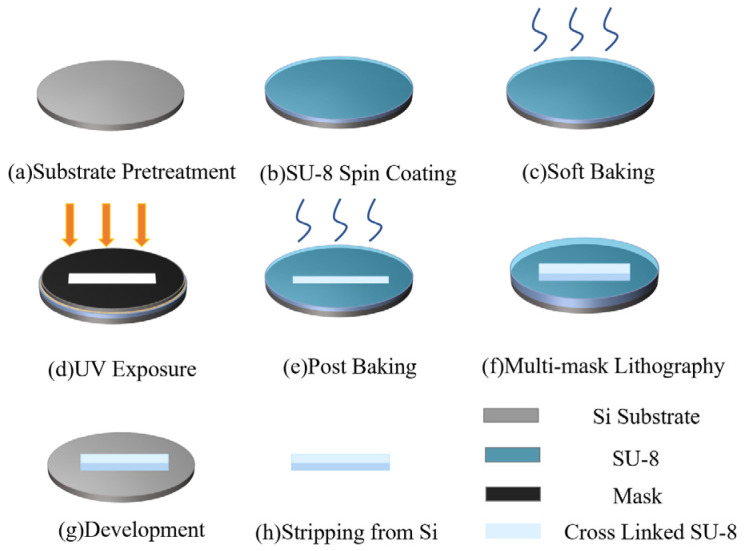
Manufacturing process of UV-LIGA technology.

The UV-LIGA process was used to prepare the cavity structure. The fabrication process steps shown are as follows [24,25]: Firstly, a silicon chip was adopted as the substrate to evenly apply SU-8(KAYAKU MICROCHEM Company, Hong Kong, China). The thickness of the SU-8 layer in this process controlled the speed and operation time of the spin coater. After spinning, the SU-8 layer is completed; soft baking was executed in Figure 9c. UV exposure is performed in Figure 9d after the sample has been soft-baked. The temperature, time of the soft baking process, and the ultraviolet light dose are determined using the thickness of the SU-8 layer. Finally, the post baking was completed. The processing steps for each SU-8 layer are the same as described above. Following the completion of all operations, the sample was developed and stripped from the silicon chip. The cross-linked SU-8 layers form the structure.

The proposed Lens antenna was machined in four parts: the part of cavity, cover plate, Lens array, and the radome. The processing procedures of the cavity structure in Figure 9 are as follows: the 300 μm thick bottom plate of the cavity structure was firstly made using SU-8. The side walls were finished using three steps: the front two steps are 600 μm high and the last step is 70 μm high. All lays were fabricated using SU-8, sequentially. The cover plate was made by using a 300 μm thick SU-8, simultaneously. After development and stripping from the substrate, sputter the metal to the sample. In order to ensure the transmission of electromagnetic waves in the cavity, the surface metal sputtering of the produced sample was required. According to Formula (10), the skin depths of copper film, with resistivity of 1.678 × 10^−8^ Ω/m, are about 230 nm at 100 GHz, respectively. To reduce the loss of electromagnetic waves, the sputtering thickness of the copper film should be at least 3δ [26]. Finally, the sample was sputtered with a metal layer of 1 µm.

(10) $$\delta =\sqrt{\frac{\rho }{\pi f\mu }}$$

where δ is the skin depth of the conductor, *ρ* is the resistivity, *f* is the working frequency, and *μ* is the absolute magnetic permeability.

**Figure 10:**
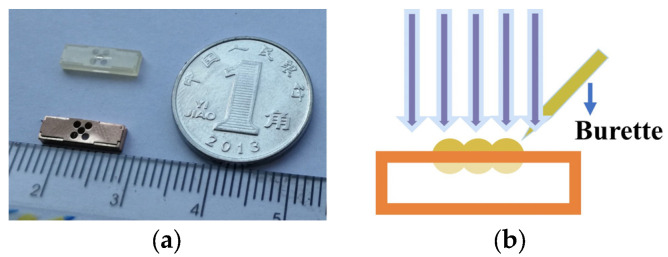
Fabricated structure of the Lens antenna: (**a**) The cavity antenna structure; (**b**) The process of making the Lens.

After the cavity and cover plate were fabricated, the cover plate was then sealed on top of the main structure to complete the fabrication of the antenna. The image of the cavity antenna structure is shown in Figure 10a. The Lens array was fabricated using UV cure adhesive (NOA73), which offers the function of focusing. The process of making the Lens array is shown in Figure 10b. NOA73 lenses were shaped using a small dose of burette under a microscopically and were then exposed under the UV light. The NOA73 Lens have the maximum absorption of ultraviolet light when using the wavelength of 350–380 nm. The energy of ultraviolet light is about 4 J/cm^2^. As observed from the image of Laser Confocal Microscope, the microstructure shows excellent sidewall quality and low surface roughness. The preparation of the Lens radome requires a 3D printing process to process its negative mold structure; then, use NOA73 for mold preparation.

**Figure 11:**
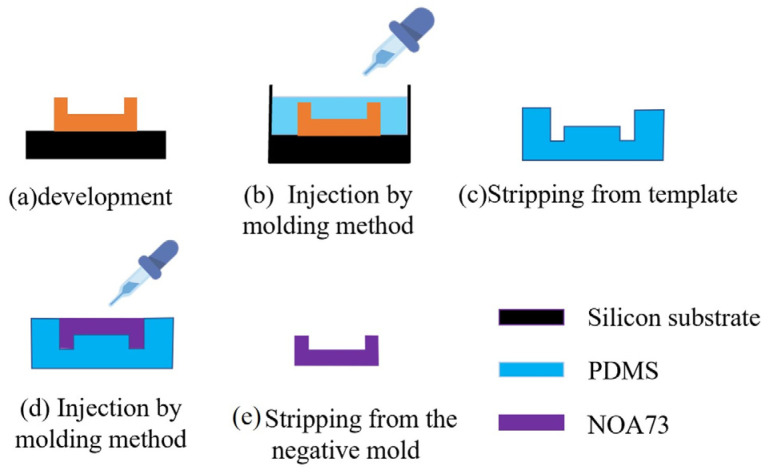
Rapid duplication of the sample.

The MEMS technology also produces a negative mode with PDMS in Figure 11, so that the cavity can be duplicated quickly. Duplication of the materials can adopt NOA73 similarly. This method reduces the processing cost and improves the processing efficiency to a certain extent.

## 3. Result and Discussion

The designed Lens antenna is fabricated using the UV-LIGA process method with the whole size of 1.87 mm high, 3.54 mm wide, and 8.6 mm length. In order to measure the reflection coefficient of the all-optical polymer Lens antenna, the network analyzer AV3672E and spread spectrum module AV3645A are used to measure the Lens antenna via Line–Reflect–Line (LRL) calibration.

**Figure 12:**
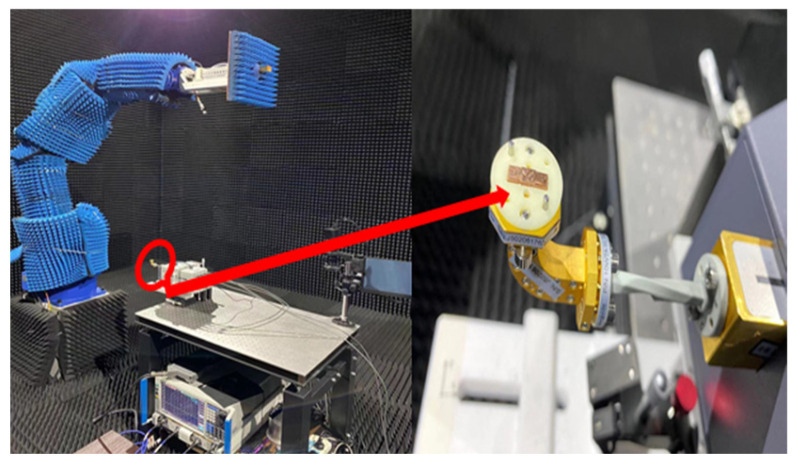
The measurement platform.

**Figure 13:**
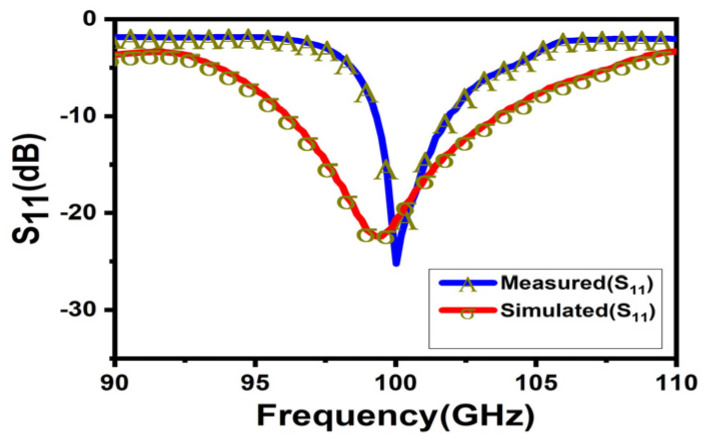
The reflection coefficient of the waveguide antenna.

**Figure 14:**
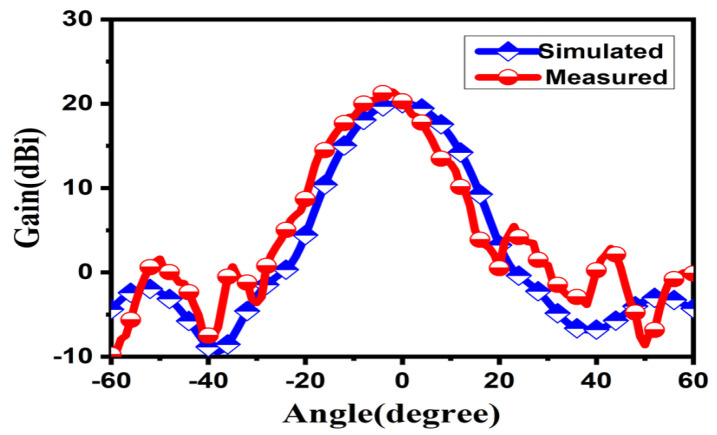
The Simulated and Measured results of Lens antenna.

Figure 12 shows the bare WR-10 waveguide antenna mounted on the WR-10 feeding port connected to the expansion module. In Figure 13, the reflection coefficient firstly reduces with aggrandizing frequency and then one relaxation peak appears at frequencies around 100 GHz. Compared with the result of simulated reflection coefficient, the result of the measured reflection coefficient has shown a narrow bandwidth and the center frequency is shifted a little to the right. The deviation in center frequency and bandwidth was mainly caused by the micromachining error and measurement error which are within a reasonable range. One of the reasons for the error is the sample height error caused by the inability to accurately control the thickness of the SU-8 during processing. In addition, the incorrect connection between the feed port and the spread spectrum module will lead to a large measurement error. The realized gain at 0° in the passband has been plotted in Figure 14. It shows a slightly skewed radiation pattern towards the top of the antenna. The measurement results have shown that the antenna design achieves the antenna gain at 0° of probably 22 dBi over the operating band and the fabricated Lens antenna exhibits an excellent pattern of symmetrical, clean, and low sidelobes.

Furthermore, we have compared the antenna with published waveguide slot array antennas in recent years with a similar working frequency. The performance index of those antennas has been summarized in Table 1. The antennas in Refs. [3,27,28,29] have higher radiation gain by adopting antenna arrays, but the total size is to big due to the very large number of elements leading to more complex design and simulation. The antenna in this work adopts a Lens radome which has reduced the complexity of the design. In addition, the antenna in this work processed using UV-LIGA technology has the advantages of light weight, high processing accuracy, and reasonable price, which basically solves some problems existing in other processes. According to the comparison in Table 1, the performance of the antenna in this work is feasible and, more importantly, meets the light-weight and miniaturization demands of wireless communication system.

## 4. Conclusions

This paper presents a novel Lens antenna based on an electromagnetic focusing Lens fabricated using NOA73 and an antenna radome. The UV-LIGA technology was adopted for processing and the feasibility of the design was verified through measurement. The device provides a high gain of 22 dBi, which can be extended to other frequency bands in the THz range by adjusting the radius of the Lens. This device is compatible with high data rate transmissions, establishing its feasibility for use in future wireless communication systems.

## Figures and Tables

**Table 1:** Antenna performance comparison.

Ref.	Antenna Type	Frequency (GHz)	Size (mm^3^)	Max Gain (dBi)	Preparation Technology
[27]	Waveguide slotted array antenna	91.5–94.5	36 × 32 × 8	25.9	Milling process
[3]	SIC slotted antenna	81.2–86	20.8 × 20.8 × 1.1	25.3	PCB process
[28]	Waveguide slotted array antenna	85–105	20 × 27 × 7	26.8	Electroforming process
[29]	Gap waveguide array antenna	89.9–97.6	55 × 55 × 9	30	Milling process
This work	Waveguide Lens antenna	97.5–104	16 × 16 × 9	22	UV-LIGA technology

## Data Availability

The data are real and shareable.
